# Development of an miRNA-Array-Based Diagnostic Signature for Periodontitis

**DOI:** 10.3389/fgene.2020.577585

**Published:** 2020-12-16

**Authors:** Su-Han Jin, Jian-Guo Zhou, Xiao-Yan Guan, Guo-Hui Bai, Jian-Guo Liu, Liang-Wen Chen

**Affiliations:** ^1^Department of Orthodontics, Affiliated Stomatological Hospital of Zunyi Medical University, Zunyi, China; ^2^Department of Oncology, Affiliated Hospital of Zunyi Medical University, Zunyi, China; ^3^School of Stomatology, Zunyi Medical University, Zunyi, China; ^4^Special Key Laboratory of Oral Diseases Research, Higher Education Institution, Zunyi, China; ^5^Hubei-MOST KLOS & KLOBM, Department of Oral Implantology, School and Hospital of Stomatology, Wuhan University, Wuhan, China

**Keywords:** periodontitis, miRNA, diagnostic signature, LASSO method, ROC curve

## Abstract

Periodontitis progression is accompanied by irreversible alveolar bone absorption and leads to tooth loss. Early diagnosis is important for tooth stability and periodontal tissue preservation. However, there is no recognized miRNA diagnostic signature with convincing sensitivity and specificity for periodontitis. In this study, we obtained miRNA array expression profiles of periodontitis from the Gene Expression Omnibus (GEO) database. After screening for differentially expressed miRNAs, the least absolute shrinkage and selection operator (LASSO) method was performed to identify and construct a 17-miRNA-based diagnostic signature (hsa-miR-3917, hsa-mir-4271, hsa-miR-3156, hsa-miR-3141, hsa-miR-1246, hsa-miR-125a-5p, hsa-miR-671-5p, hcmv-mir-UL70, hsa-miR-650, hsa-miR-497-3p, hsa-miR-145-3p, hsa-miR-141-3p, hsa-miR-210-3p, hsa-miR-204-3p, hsa-miR-203a-5p, hsa-miR-99a-3p, and hsa-miR-30a-3p). Periodontal tissue samples with higher risk scores were more likely to show symptoms of periodontitis. Then, the receiver operating characteristic (ROC) curves were used to assess the diagnostic value of the miRNA signature, which indicated that the optimum cutoff value in periodontitis diagnosis was 0.5056 with an area under the ROC curve (AUC) of 0.996, a sensitivity of 97.3%, a specificity of 100.0% in the training cohort; in the testing cohort, the corresponding values were as follows: an AUC of 0.998, a sensitivity of 97.9%, and a specificity of 91.7%. We next evaluated the efficacy of the signature in differentiating disease subtype and affected range. Furthermore, we conducted functional enrichment analysis of the 17 miRNA-targeted mRNAs, including the regulation of mTOR activity and cell autophagy, Th1/Th2 cell balance and immunoregulation, cell apoptosis, and so on. In summary, our study identified and validated a 17-miRNA diagnostic signature with convincing AUC, sensitivity, and specificity for periodontitis.

## Introduction

Periodontitis, reported as one of the most prevalent diseases worldwide (Kassebaum et al., 2014), begins with damage to periodontal tissue, with typical symptoms, including gingival recession, attachment loss, and alveolar bone resorption, and eventually leads to tooth loss (Gross et al., 2017; Papapanou and Susin, 2017). Tooth loss from aggressive periodontitis (AP) is 0.05–0.12 per patient-year (Nibali et al., 2013; Clark et al., 2017). Periodontitis identified at an early stage carries a much-improved prognosis compared to advanced-stage disease because of the irreversible alveolar bone absorption that occurs during progression (Hienz et al., 2015). Thus, the early diagnosis of periodontitis is crucial for alveolar bone preservation and tooth stability.

At present, numerous inflammatory cytokines, such as proinflammatory cytokines, tumor necrosis factor, anti-inflammatory cytokines, and host factor matrix metalloproteinase, are considered as biomarkers for periodontitis (Cafiero et al., 2013; Kinney et al., 2014; Luan et al., 2018). However, there are limited reports on non-coding RNAs as biomarkers for periodontitis. Recently, miRNAs have emerged as a novel class of highly sensitive and specific biomarkers and have been reported to play critical regulatory roles in periodontal homeostasis and during periodontitis progression (Sugatani and Hruska, 2007; Hung et al., 2010; Liu et al., 2011; Chen et al., 2014; Guo et al., 2014; Zhou et al., 2016; Luan et al., 2018). Thus, miRNAs are promising biomarkers for periodontitis diagnosis or prognosis (Schmalz et al., 2016; Saito et al., 2017; Luan et al., 2018; Mico-Martinez et al., 2018).

A variety of miRNAs are dysregulated in periodontitis tissue compared to healthy tissue, such as upregulated miR-15a, miR-29b, miR-125a, miR-146a, miR-148/148a, and miR-223 and downregulated miR-92 (Luan et al., 2018). However, there is no recognized miRNA diagnostic signature for periodontitis, with convincing sensitivity and specificity, until now (Arias-Bujanda et al., 2019). In this study, we compared the miRNA profiles between disease and control samples and identified an miRNA diagnostic signature with high sensitivity and specificity for periodontitis.

## Materials and Methods

### Search Strategy

The Gene Expression Omnibus (GEO^1^) database was searched to find datasets related to periodontitis, using “Homo sapiens [porgn: txid9606] and periodontitis”; 14 datasets met the criteria. Only five datasets collected miRNA profiles. To reduce random error, we limited the target to datasets with a sample size greater than 100, and eventually GSE54710 was screened out.

### Sample Clinical Characteristics and Data Processing

The clinical characteristics and miRNA profiling data of periodontitis samples (GSE54710), obtained with whole-genome microarray analysis (U-133 Plus 2.0; Affymetrix, Santa Clara, CA, United States), were downloaded from the GEO database.

The data contained 159 disease samples (gingival samples showing periodontitis, with attachment level ≥3 mm) and 41 control samples (healthy gingival samples, with attachment level ≤2 mm) (Stoecklin-Wasmer et al., 2012). The miRNA microarray data were annotated with GPL15159. All miRNAs differentially expressed between the disease and control samples were analyzed using the Linear Models for Microarray data (Limma, version 3.30.0) package in R. MicroRNAs with the parameters absolute log2 fold change >0.45 and *P* value < 0.05 (Zhang et al., 2015) were regarded as differently expressed miRNAs (DE-miRNAs). The DE-miRNA expression value was log2 transformed for next data processing.

### Construction and Validation of the miRNA-Array-Based Diagnostic Model

The outcome measure was periodontitis/control classification. Two hundred samples were randomly divided into a training cohort (112 disease and 29 control samples) and a testing cohort (47 disease and 12 control samples) at a ratio of 7:3.

Afterward, we used least absolute shrinkage and selection operator (LASSO) regression analysis for DE-miRNA selection and reduction using the *glmnet* package (version 3.0) (Gao et al., 2010) in R. This LASSO method shrinks coefficients toward zero, and eliminates unimportant terms entirely, thus reducing prediction error and minimizing overfitting. The sensitivity and specificity were evaluated for each DE-miRNA and were validated within the internal validation samples. Afterward, diagnosis-associated DE-miRNAs with nonzero coefficients were selected to build a diagnostic miRNA signature. We obtained diagnostic models according to a variety of evaluation variables, all with accuracy ≥ 0.95, sensitivity ≥ 0.95, specificity ≥ 0.9, and recall ≥ 0.9. DE-miRNA-based receiver operating characteristic (ROC) curves were constructed to assess the optimal signature model in the training set with the *pROC* (version 1.15.3) package. The ROC figure was plotted using the *ggplot2* and *ggfortify* packages. Area under the ROC curve (AUC) was calculated to quantify the diagnostic accuracy (Sauerbrei et al., 2007) of the signature. Then, the cutoff value was identified according to the ROC curve, using the *local maxima* method of the *pROC* package. Thereafter, the diagnostic value of the signature was further validated in the testing set (Xu et al., 2017; Liu et al., 2019).

### The Diagnostic Signature Diagnosed Clinical Outcomes

To further explore the prognostic function of the signature, we utilized the miRNA signature for assessing the disease subtype and affected range. According to the clinical features of different subtypes, all samples were divided into AP and chronic periodontitis (CP). AP is a subtype of periodontitis with a prevalence of 0.6–2.2% (Ababneh et al., 2012; Catunda et al., 2019) and is characterized by rapid periodontal attachment loss and bone resorption (Susin et al., 2014). According to the number of affected teeth, the samples were grouped into localized periodontitis with gingival attachment loss and bone resorption sites ≤30% of all teeth, and generalized periodontitis with affected tooth sites >30%. The risk score was calculated for each sample and used to classify each sample into a high expression group (with risk scores above the cutoff value) and a low expression group (with risk scores below the cutoff value). The associations between risk scores and various clinical characteristics were analyzed with the chi-square test (Yao et al., 2018).

### Functional Enrichment of the DE-miRNA Signature

The functions of the DE-miRNAs of the diagnostic signature were explored using the competing endogenous RNA (ceRNA; lncRNA–miRNA–mRNA) network. The sequences of the identified DE-miRNAs were obtained from Ensembl and put into miRcode^2^ and starBase v 2.0^3^ to predict their lncRNA targets. To improve the data reliability, lncRNAs that have not been annotated by GENCODE^4^ were omitted. Furthermore, the mRNAs shown to be targeted by miRNAs with experimental support were then identified using miRTarBase^5^. Only miRNAs with target RNAs in these four databases were used to construct the ceRNA network. We built the ceRNA network based on the abovementioned data and used the *ggalluvial* (version 0.9.1) package to visualize the network (Jin et al., 2019).

### Gene Ontology and Kyoto Encyclopedia of Genes and Genomes Analysis

To gain further insights into the biological pathways involved in the miRNA diagnostic signature, we conducted Gene Ontology (GO) and Kyoto Encyclopedia of Genes and Genomes (KEGG) pathway analysis. An original list of the DE-mRNAs of the corresponding DE-miRNAs was predicted through miRTarBase. In addition, we obtained the differentially expressed mRNAs for periodontitis by analyzing the GSE16134 in our previous work (Jin et al., 2019). In order to appropriately restrict the number of DE-mRNAs for periodontitis, the original list of DE-mRNAs was overlapped with the differentially expressed mRNAs from GSE16134; the overlapping mRNAs between these two cohorts are shown in Supplementary Table 3. We then annotated the overlapping mRNAs using GO and KEGG pathway enrichment analysis with the *clusterProfiler* package (Ma et al., 2019) in R.

### Statistical Analysis

The sensitivity, specificity, and AUC values of the diagnostic signature were quantified based on the ROC curve. The association between the miRNA signature risk scores and categorical clinical characteristics was analyzed with the chi-square test. All statistical tests were two-sided, and *P* < 0.05 represented statistically significant results. Statistical analyses were performed with R (version 3.5.2^6^).

## Results

### Differential Expression of miRNAs

**FIGURE 1:**
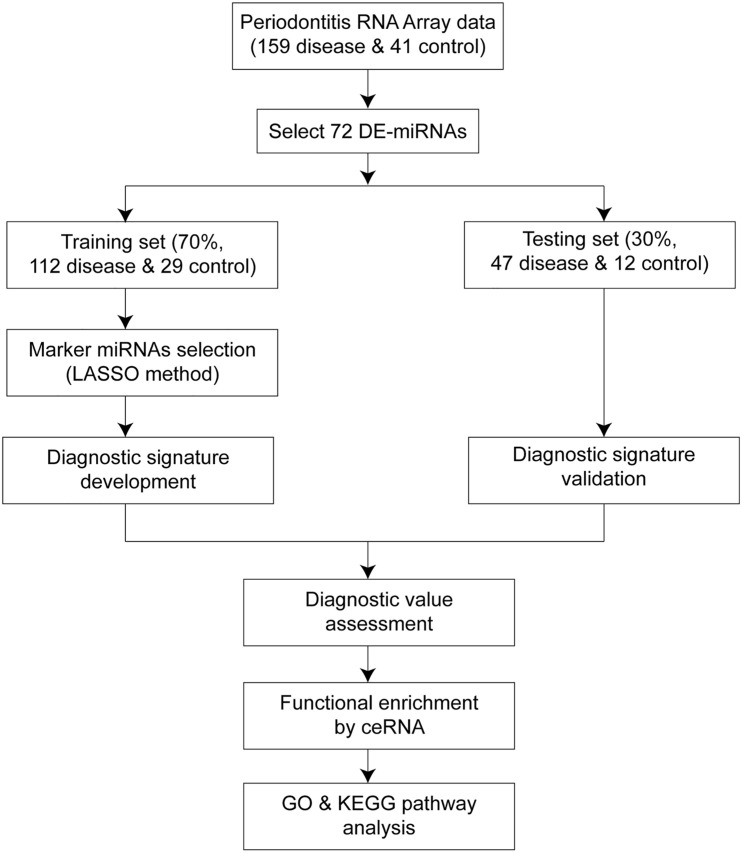
Workflow of the whole study. The overall process of diagnostic signature development, validation, assessment, and functional analysis.

**FIGURE 2:**
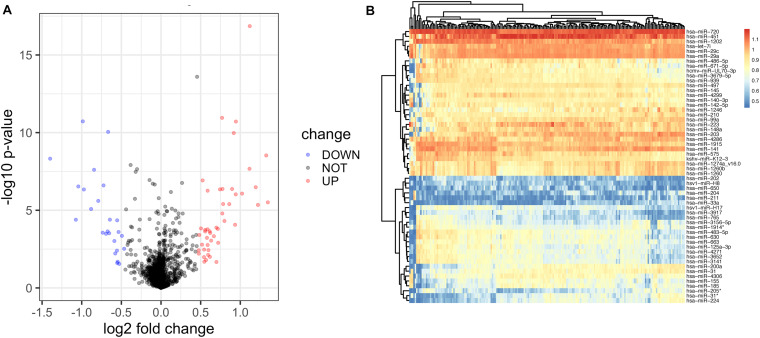
Identification of differentially expressed miRNAs of diseased gingiva from control samples using the miRNA array. **(A)** The volcano map of all DE-miRNAs. **(B)** The hierarchical clustering heat map of the 72 significant DE-miRNAs, including 47 upregulated and 25 downregulated ones.

We initially compared the miRNA profiles of 159 disease samples with 41 control samples (Figure 1). A total of 72 miRNAs, including 47 upregulated and 25 downregulated, were identified as significantly differentially expressed between the disease and control samples (Supplementary Table 1). The relative expression values of all the DE-miRNAs (Figure 2A) and the 72 significantly DE-miRNAs (Figure 2B) are illustrated, demonstrating substantial variation. The 200 samples were randomly divided into the training set (112 disease and 29 control samples) and testing set (47 disease and 12 control samples). A LASSO regression analysis was used to select and reduce variables within the 72 DE-miRNAs in the training cohort. Seventeen miRNAs were identified as variables.

### Diagnostic Signature Model Development and Validation

Next, the best risk coefficients for these miRNAs were calculated using the multivariable logistic regression method, resulting in a diagnostic signature. Our diagnostic signature obtained from the training cohort was used to establish the following formula: risk score = (0.3348278 × hsa-miR-3917) + (3.6230776 × hsa-mir-4271) + (−4.3906260 × hsa-miR-3156) + (0.1870440 × hsa-miR-3141) + (1.0495169 × hsa-miR-1246) + (1.8215707 × hsa-miR-125a-5p) + (2.1134420 × hsa-miR-671-5p) + (1.3936642 × hcmv-mir-UL70) + (0.8620107 × hsa-miR-650) + (8.5144859 × hsa-miR-497) + (6.2927132 × hsa-miR-145) + (−5.1714717 × hsa-miR-141) + (−1.4265602 × hsa-miR-210) + (−2.8905885 × hsa-miR-204) + (−7.4759805 × hsa-miR-203a) + (−3.9084222 × hsa-miR-99a) + (0.7008474 × hsa-miR-30a) (Table 1). In this signature, miR-3156, miR-141, miR-210, miR-204, miR-203a, and miR-99a have negative coefficients, while others have positive coefficients. As shown in the results, a further high signature score was diagnostic of periodontitis in the training cohort (*P* = 1.508 × 10^–36^; Table 1).

**TABLE 1:** Eleven miRNAs used in the diagnostic signature for periodontitis prediction.

Probe ID	Mature_sequence	Diagnostic signature coefficient	logFC	*P* value	Adj. *P* value	Change
MIMAT0018191	hsa-miR-3917	0.3348278	1.119281209	1.386256585 × 10^–17^	1.868673876 × 10^–14^	UP
MIMAT0016901	hsa-mir-4271	3.6230776	0.620785324	0.000288612	0.004471832	UP
MIMAT0015030	hsa-miR-3156	–4.3906260	–0.791522355	2.486364974 × 10^–6^	0.000101564	DOWN
MIMAT0015010	hsa-miR-3141	0.1870440	0.666519204	0.000467033	0.006295600	UP
MIMAT0005898	hsa-miR-1246	1.0495169	–1.398413629	4.753978518 × 10^–9^	7.120403381 × 10^–7^	DOWN
MIMAT0004602	hsa-miR-125a-5p	1.8215707	0.698621543	0.000131228	0.002640229	UP
MIMAT0003880	hsa-miR-671-5p	2.1134420	1.194737651	3.278450055 × 10^–7^	0.000020088	UP
MIMAT0003343	hcmv-mir-UL70	1.3936642	1.115945994	2.130063583 × 10^–8^	2.130063583 × 10^–8^	UP
MIMAT0003320	hsa-miR-650	0.8620107	0.770481030	1.116784747 × 10^–11^	5.018086129 × 10^–9^	UP
MIMAT0002820	hsa-miR-497	8.5144859	0.632080330	0.000898469	0.011111338	UP
MIMAT0000437	hsa-miR-145	6.2927132	0.944161897	1.930765232 × 10^–1^	5.205343065 × 10^–9^	UP
MIMAT0000432	hsa-miR-141	–5.1714717	–0.881768391	8.288353384 × 10^–6^	0.000279318	DOWN
MIMAT0000267	hsa-miR-210	–1.4265602	–0.986430233	1.886156327 × 10^–11^	5.205343065 × 10^–9^	DOWN
MIMAT0000265	hsa-miR-204	–2.8905885	–0.644798765	0.000040156	0.000040156	DOWN
MIMAT0000264	hsa-miR-203a	–7.4759805	–1.074061110	0.000040611	0.000960419	DOWN
MIMAT0000097	hsa-miR-99a	–3.9084222	–0.735644039	0.000277329	0.004398111	DOWN
MIMAT0000087	hsa-miR-30a	0.7008474	0.483432172	0.000391601	0.005442038	UP

**FIGURE 3:**
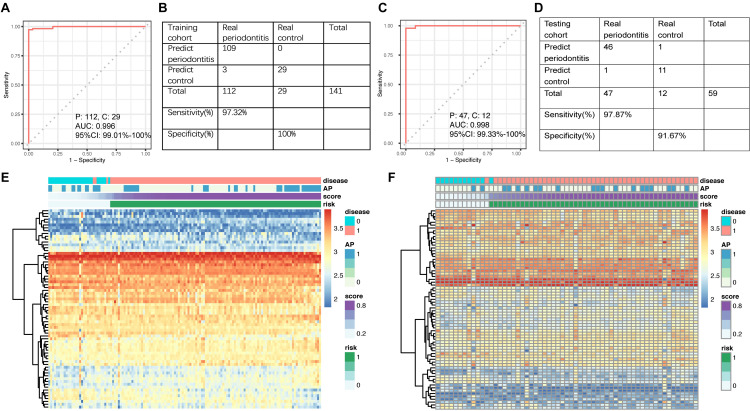
**(A)** ROC curve of the diagnostic signature in the training set. **(B)** Confusion table of binary results of the diagnostic signature in the training set. **(C)** ROC curve of the diagnostic signature in the testing set. **(D)** Confusion table of binary results of the diagnostic signature in the testing set. Unsupervised hierarchical clustering of 17 miRNA markers selected for use in the diagnostic signature in the **(E)** training and **(F)** testing set.

In the training set, the AUC of the signature for periodontitis diagnosis was 0.996 (95% CI: 0.990–1.000; Figure 3A). The ROC curve was used to determine the best cutoff value in periodontitis diagnosis, which was 0.5056. Applying the miRNA signature yielded a sensitivity of 97.3% and a specificity of 100.0% in the training dataset (Figure 3B). To validate the diagnostic value of the signature in the testing cohort, we used the equation above to compute risk scores with the clinical outcome and miRNA profile data. The distribution of risk scores for the testing set was very similar to that of the training set. In the testing set, the area under the ROC curve for the signature was 0.998 (95% CI: 0.993–1.000; Figure 3C). Applying the miRNA signature yielded a sensitivity of 97.9% and a specificity of 91.7% (Figure 3D). The 17 miRNAs in the training set (Figure 3E) and testing set (Figure 3F) were able to distinguish periodontally diseased gingiva from normal controls.

### The Diagnostic Signature Diagnosed Clinical Outcomes

**FIGURE 4:**
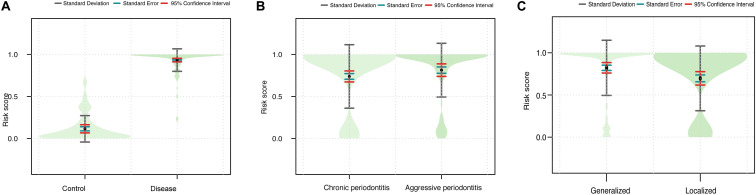
Violin plots showing the relationship between the signature and the clinical outcomes. **(A)** The combined risk score in the disease vs. control group. **(B)** The combined risk score in the CP vs. AP group. **(C)** The combined risk score in the generalized vs. localized group.

We next assessed the efficacy of the miRNA signature in differentiating control vs. disease samples, CP vs. AP, and localized vs. generalized range. The risk scores were calculated for each sample. Then, the cutoff value was used to divide 200 samples into two groups: 156 cases showed high risk scores, and 44 showed low risk scores (Table 2). The diagnostic signature could differentiate disease samples from control samples (*P* = 1.508 × 10^–36^, Figure 4A), as well as the affected range of periodontitis (*P* = 0.015, Figure 4C). In general, periodontal tissue samples with high risk scores were more likely to show symptoms of periodontitis. In contrast, the disease subtype (*P* = 0.138, Figure 4B) was not significantly associated with the signature scores.

**TABLE 2:** Association of the diagnostic signature risk scores with clinical characteristics.

Clinical features	Cases	Diagnostic signature risk scores		*P* value
		High	Low	
Clinical state	200	156	44	1.508 × 10^–36^
Diseased	159	155	4	
Control	41	1	40	
Disease subtype				0.138
CP	128	96	32	
AP	72	60	12	
Affected range				0.015
Generalized	109	90	19	
Localized	91	66	25	

### Functional Enrichment of the DE-miRNA Signature

**FIGURE 5:**
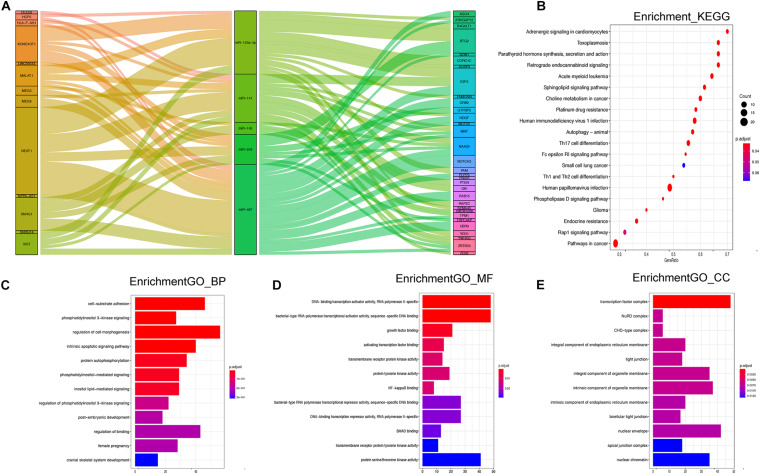
**(A)** Sankey diagram for the ceRNA network analysis. Each rectangle represents a gene, and the connection degree of each gene is visualized based on the size of the rectangle. **(B)** Top 20 KEGG signaling pathway enrichment of the 17 miRNA-targeted mRNAs. **(C–E)** Top 12 GO functional enrichment of the 17 miRNA-targeted mRNAs.

The 17 DE-miRNAs of the signature were put into the miRcode and starBase v 2.0 databases to predict their lncRNA targets. Then, the corresponding coding genes of the DE-miRNAs were predicted through miRTarBase. After removing results that did not meet the screening criteria, 5 miRNAs, 13 lncRNAs, and 32 mRNAs were used to establish a ceRNA network (Figure 5A). The detailed data of all 81 ceRNA network components are shown in Supplementary Table 2. Furthermore, the degree of connection of each gene by topology was calculated to illustrate its importance in the ceRNA network (Figure 5A).

### GO and KEGG Analysis

There were 1,107 overlapping mRNAs between the previously mentioned two cohorts (Supplementary Table 3). Functional enrichment analysis identified 441 GO terms in the biological process (BP) category, 19 GO terms in the cellular component (CC) category, 16 GO terms in the molecular function (MF) category, and 48 KEGG pathways (Supplementary Table 4). The top 12 GO results and top 20 KEGG results are shown in Figure 5. Regarding BP, the DE-mRNAs were involved in cell–substrate adhesion, the intrinsic apoptotic signaling pathway, and regulation of cell morphogenesis (Figure 5C). In terms of MF, the DE-mRNAs were mainly associated with DNA-binding transcription activator activity, bacterial-type RNA polymerase transcriptional activator activity, and bacterial-type RNA polymerase transcriptional repressor activity, among other associations. The CCs of the DE-mRNAs were transcription factor complex, nuclear envelope, intrinsic component of organelle membrane, and so on. In addition, the KEGG results showed enrichment in the rap 1 signaling pathway, phospholipase D signaling pathway, autophagy animal, and Th1 and Th2 cell differentiation, and so on.

## Discussion

The early diagnosis of periodontitis helps to preserve teeth and improve quality of life. However, clinical monitoring is time-consuming, is subject to considerable measurement error, and is often poorly tolerated by patients (Miller et al., 2010; Kinane et al., 2017). Thus, objectively quantifiable biomarkers are needed for periodontitis diagnosis.

For single molecules, the highest reported values of sensitivity were obtained by IL-1β (78.7%) (De Luca Canto et al., 2015), MMP-8 (75.5%) (Alassiri et al., 2018), IL-6 (72%) (Lee et al., 2018), and hemoglobin (72%) (Nomura et al., 2016). The highest reported values of specificity were obtained by IL-1β (78.0%) and MMP-9 (77.0%) (Kim et al., 2016). For the combination diagnostic models, the combination of IL-1β, IL-1ra, and MMP exhibited the highest AUC (0.853) with high sensitivity (73.3%) and specificity (88.9%) (Wu et al., 2018). The TdPiTfAaFnPm bacterial cluster-based model has an AUC ≥ 0.787 with a sensitivity and specificity ≥ 80.0% (Tomas et al., 2017). However, there are limited reports on non-coding RNAs as biomarkers for periodontitis.

Previous studies have highlighted a single miRNA biomarker of periodontitis, such as miR-143-3p (*P* ≤ 0.05) (Nisha et al., 2019) or miR-1226 (*P* < 0.05) (Mico-Martinez et al., 2018). However, the analysis of single miRNAs cannot provide a complete picture of the existing physiological and pathological state. Multi-miRNA signatures may have higher AUC, sensitivity, and specificity values. Therefore, for the first time, we developed and validated a multiple miRNA-based diagnostic signature for periodontitis, obtaining an AUC of 0.996 in the training set and an AUC of 0.998 in the testing set, which is very convincing for periodontitis biomarkers, to the best of our knowledge.

These results demonstrate that the signature can effectively assess disease vs. control. The signature also can differentiate affected range, which may be because these two diagnoses have similarities. The main criterion of affected range is affected tooth number. Thus, the more tooth positions show symptoms of periodontitis, the higher the risk score will be. However, the signature cannot assess AP vs. CP, which may be because both AP and CP belong to disease samples, and hence cannot be distinguished effectively.

The signaling pathway enrichment analysis indicated that the 17 miRNA-targeted mRNAs play important roles in the rap 1 signaling pathway, phospholipase D signaling pathway, and autophagy animals, which are closely related to the regulation of mTOR activity and cell autophagy (Liu et al., 2013; Zhao et al., 2015; Chen et al., 2018). The other enriched pathways include Th1 and Th2 cell differentiation and Th17 cell differentiation; these pathways are directly connected to Th1/Th2 balance and immunoregulation (Yun et al., 2003; Orozco et al., 2007). In addition, the Fc epsilon RI signaling pathway induces NF-kappa B activation in mast cells and mast cell degranulation (Klemm and Ruland, 2006), which is related to cell apoptosis. Their involvement with pathways in cancer confirmed an association between various types of cancer and clinical signs of periodontitis, combined with poor oral health (Hoare et al., 2019).

There are limitations to our research. Although the AUC, sensitivity, and specificity of the diagnostic signature are convincing for non-coding RNA-based signatures, the samples came from periodontal gingival tissue, which is difficult to collect. Further research should focus on miRNA-based diagnostic signatures of gingival crevicular fluid for periodontitis. Besides, available data are not enough for external verification, which needs future work to be perfected.

## Conclusion

Our study identified a 17-miRNA-based diagnostic signature for periodontitis, and it warrants large-scale clinical validation in the future.

## Data Availability Statement

The dataset presented in this study can be found in online repositories. The names of the repository/repositories and accession number(s) can be found in the article/ Supplementary Material.

## Author Contributions

S-HJ drafted the manuscript. J-GZ analyzed the data. X-YG and G-HB interpreted the data. J-GL revised the work critically. L-WC drafted the manuscript and gave final approval of the version to be published. All authors had made substantial contributions to conception and design of the study.

## Conflict of Interest

The authors declare that the research was conducted in the absence of any commercial or financial relationships that could be construed as a potential conflict of interest.

## Footnotes

1 https://www.ncbi.nlm.nih.gov/geo/

2 http://www.mircode.org/

3 http://starbase.sysu.edu.cn/

4 http://www.gencodegenes.org/

5 http://mirtarbase.mbc.nctu.edu.tw/

6 http://www.Rproject.org

## Supplementary Material

Supplementary Figure 1 Violin plots showing the relationship between 17 miRNAs selected for use in the diagnostic signature and the clinical outcomes.

Supplementary Table 1 Comparison of patient characteristics between diseased and control samples within 72 DE-miRNAs.

Supplementary Table 2 The detailed data of all ceRNA network components.

Supplementary Table 3 The detailed data of overlapping DE-mRNAs.

Supplementary Table 4 The functional enrichment assay identified 441 GO terms in BP, 19 GO terms in CC, 16 GO terms in MF, and 48 KEGG pathway.
