# Supplementary figures and images for: Development of an miRNA-Array-Based Diagnostic Signature for Periodontitis
Source: Front Genet. 2020 Dec 16;11:577585. doi: 10.3389/fgene.2020.577585 (PMC7772397; doi:10.3389/fgene.2020.577585)

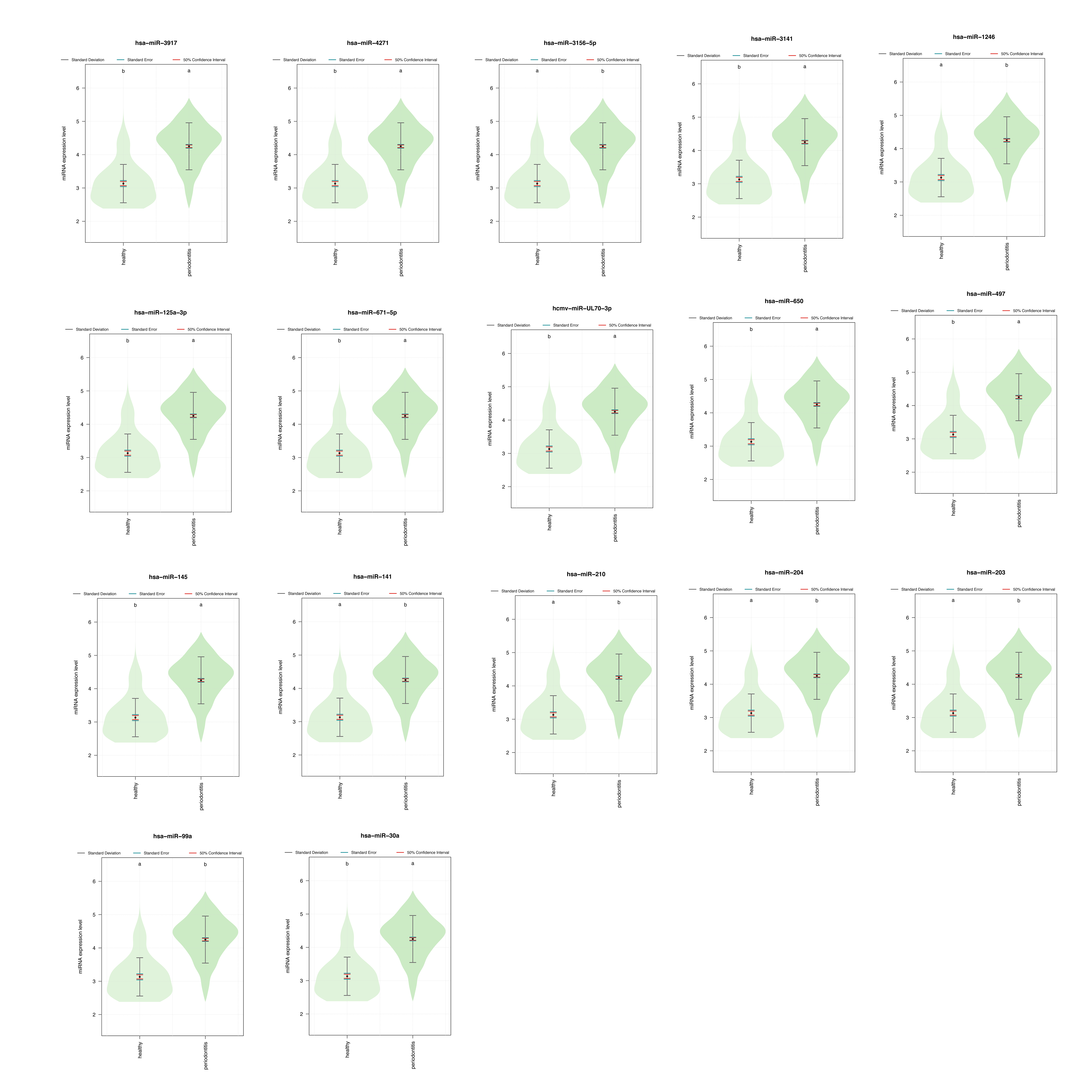

Supplement: Supplementary Figure 1 — Violin plots showing the relationship between 17 miRNAs selected for use in the diagnostic signature and the clinical outcomes. [file Image_1.TIF]
